# Precision Targeted Therapy for PCOS: Emerging Drugs, Translational Challenges, and Future Opportunities

**DOI:** 10.3390/biomedicines14010213

**Published:** 2026-01-19

**Authors:** Xinhong Wu, Wei Yi, Xiawen Liu

**Affiliations:** Guangdong Provincial Key Laboratory of Molecular Target and Clinical Pharmacology, The State Key Laboratory of Respiratory Disease, School of Pharmaceutical Sciences, Guangzhou Medical University, Guangzhou 511436, China; 19838927929@163.com

**Keywords:** polycystic ovary syndrome (PCOS), hyperandrogenism (HA), insulin resistance (IR), precision medicine, network pharmacology

## Abstract

Polycystic Ovary Syndrome (PCOS) is characterized by a self-perpetuating vicious cycle between insulin resistance (IR) and hyperandrogenism (HA). While lifestyle management remains the internationally recommended first-line treatment, current clinical management, primarily relying on combined oral contraceptives and metformin, offers symptomatic relief or “masking” of the phenotype but fails to adequately disrupt this core pathophysiological loop, while also carrying potential intergenerational safety concerns. This review systematically evaluates the paradigm shift toward mechanism-based precision medicine. First, we analyze emerging precision-targeted therapies that intervene in specific pathological nodes: (1) metabolic regulators (e.g., GLP-1RAs, SGLT2i, and brown adipose tissue (BAT) activators) that target systemic glucotoxicity and the novel “BAT-Ovarian axis”; (2) neuroendocrine modulators (e.g., NK3R antagonists) that act as negative modulators of the hyperactive GnRH pulse generator; and (3) innovative androgen synthesis inhibitors (e.g., Artemisinins) that utilize a degradation-at-source mechanism. Complementing these, we explore the strategic value of Natural Products through the lens of “Network Pharmacology”, highlighting their ability to restore systemic homeostasis via multi-target modulation. Finally, we address critical translational challenges, specifically the need to establish long-term reproductive and offspring safety, providing a roadmap for developing true disease-modifying treatments for PCOS. Distinct from reviews limited to isolated therapeutic modalities, this article uniquely bridges current clinical management with emerging organ-specific precision targets and natural product networks.

## 1. Introduction

Polycystic ovary syndrome (PCOS) represents the most prevalent endocrine-metabolic disorder in women of reproductive age, affecting 6% to 21% of the global population [1,2,3]. It is clinically defined by at least two of the following: (1) oligo-anovulation, (2) hyperandrogenism (clinical or biochemical), and (3) polycystic ovary morphology or increased anti-Müllerian hormone levels [4]. Patients face a 4- to 10-fold increased risk of Type 2 Diabetes Mellitus (T2DM), alongside heightened susceptibility to cardiovascular disease, endometrial cancer and psychological morbidities such as anxiety and depression [5,6,7].

At the pathophysiological core of PCOS lies a self-perpetuating “vicious cycle” between insulin resistance (IR) and hyperandrogenism (HA), underpinned by a state of chronic systemic inflammation. Hyperinsulinemia drives excess ovarian androgen production via direct stimulation of theca cells, while androgen excess, in turn, exacerbates metabolic dysfunction and visceral adiposity, further fueling the inflammatory milieu. Unfortunately, current clinical management strategies—primarily relying on combined oral contraceptives (COCPs) and metformin—often fail to disrupt this core cycle. As detailed in this review, these traditional therapies largely offer symptomatic relief or “masking” of the phenotype rather than disease modification, and are further limited by significant side effects and safety concerns during pregnancy.

Consequently, the landscape of PCOS drug development is undergoing a paradigm shift towards mechanism-based precision medicine. This review systematically evaluates this transition through two emerging therapeutic distinct strategies. First, we examine precision-targeted therapies that precisely intervene in specific pathological nodes, from novel metabolic regulators (e.g., GLP-1RAs, SGLT2i, and BAT activators) to neuroendocrine modulators (e.g., NK3R antagonists) and innovative androgen degraders (e.g., Artemisinins). Second, we explore the concept of “Network Pharmacology” via natural products, which utilize multi-target mechanisms to restore systemic homeostasis. By synthesizing these advances, we aim to provide a roadmap for moving beyond symptom management toward truly disease-modifying treatments.

Literature Search Methodology: A comprehensive literature search was conducted in PubMed and Web of Science databases up to December 2025. The search strategy utilized a combination of keywords including ‘PCOS’, ‘Insulin Resistance’, ‘Hyperandrogenism’, along with specific therapeutic classes (e.g., ‘GLP-1 receptor agonists’, ‘NK3R antagonists’, ‘Artemisinin’, ‘Natural Products’). To ensure the quality and relevance of cited publications, priority was given to Randomized Controlled Trials (RCTs), systematic reviews, and high-impact mechanistic studies published predominantly within the last 5–10 years, while excluding non-peer-reviewed articles and abstracts without full text.

## 2. Current Clinical Management: Efficacy and Unmet Needs

### 2.1. Lifestyle Interventions: Efficacy and Adherence Issues

Lifestyle modification, encompassing dietary restrictions (e.g., healthy, balanced diets such as low-glycemic index or Mediterranean-style diets) and physical activity, remains the universally mandated first-line therapy. Indeed, rigorous diet and exercise interventions have demonstrated a significant impact on reducing or reversing the core pathologies of IR, hyperinsulinemia, and HA. The physiological basis is robust: a weight loss of even 5% can ameliorate IR, increase Sex Hormone-Binding Globulin (SHBG), and spontaneously restore ovulation in a subset of patients [8,9,10].

However, the clinical utility is severely capped by adherence and sustainability. Long-term maintenance of weight loss is notoriously difficult, and lifestyle monotherapy is often insufficient to normalize reproductive outcomes, such as live birth rates, in patients with severe metabolic phenotypes.

### 2.2. Pharmacological Therapies

#### 2.2.1. COCPs: Mechanisms and Limitations

COCPs are first-line medications for treating menstrual disorders and cutaneous hyperandrogenism.

Mechanistically, they provide dual suppression: the progestin component inhibits the hypothalamic-pituitary axis while the estrogen component suppresses FSH secretion via negative feedback and stimulates hepatic SHBG production, thereby reducing bioavailable testosterone [4]. While COCPs effectively manage hyperandrogenism via LH suppression and SHBG elevation, their primary limitation is that they offer symptomatic management rather than disease modification, as they do not address—or may even exacerbate—the underlying IR [11,12].

Furthermore, their use requires a careful risk-benefit analysis regarding the additive risk of venous thromboembolism (VTE), particularly in patients with co-existing obesity or advanced reproductive age, who already harbor a baseline thrombotic risk due to PCOS itself [13,14,15,16].

#### 2.2.2. Insulin Sensitizers (Metformin): Clinical Outcomes and Safety

Metformin remains the most widely used insulin sensitizer, directly targeting IR—a core pathophysiological driver of PCOS. By activating the AMPK pathway, it effectively improves glucose homeostasis, lipid profiles, and menstrual regularity [17]. Beyond AMPK activation, metformin exerts pleiotropic effects, including the modulation of gut microbiota and suppression of hepatic gluconeogenesis, as detailed in Table 1. Notably, when combined with COCPs, metformin demonstrates superior efficacy in ameliorating hyperandrogenism, reducing the Free Androgen Index (FAI), and elevating SHBG levels compared to COCP monotherapy [17].

Despite its status as a foundational therapy, metformin’s clinical utility is constrained by variable clinical responses. While it demonstrates robust efficacy in improving metabolic parameters in IR phenotypes, its impact on clinical HA and reproductive outcomes in non-obese patients can be inconsistent. For ovulation induction, metformin has been superseded by letrozole due to inferior live birth rates [18,19,20,21].

Furthermore, systematic reviews indicate that it does not consistently improve clinical pregnancy or live birth rates in patients undergoing IVF/ICSI [22]. Gastrointestinal adverse events (e.g., nausea, diarrhea) are frequent, significantly hampering patient adherence. Perhaps most critically, long-term follow-up from the Metformin Interventions in Gestation (MiG) trial suggests that maternal metformin use may paradoxically increase the risk of childhood obesity and cardiovascular dysfunction in offspring, raising safety concerns regarding its use during pregnancy [23].

#### 2.2.3. Ovulation Induction Agents: Efficacy and Risks

While clomiphene citrate was historically the standard of care, letrozole (an aromatase inhibitor) has replaced it as the first-line treatment for anovulatory infertility due to superior live birth rates. Gonadotropins remain a second-line option for resistant cases. By inhibiting estrogen synthesis, Letrozole releases the hypothalamus from negative feedback, increasing FSH secretion to drive follicular development [24].

While effective for achieving pregnancy, letrozole targets the clinical consequence (anovulation) without rectifying the upstream metabolic or neuroendocrine dysregulation. Compared to other ovulation induction agents (e.g., gonadotropins), letrozole is associated with a significantly lower risk of Ovarian Hyperstimulation Syndrome (OHSS) and multiple gestations, owing to its shorter half-life and promotion of mono-follicular growth [25].

#### 2.2.4. Anti-Androgens: Efficacy and Contraindications

Spironolactone is employed for moderate-to-severe hirsutism unresponsive to COCPs. It acts by competitively blocking the androgen receptor (AR) and inhibiting 5α-reductase activity [26].

Its use presents a therapeutic paradox for women of reproductive age: due to its potential teratogenicity (risk of feminizing a male fetus), strict contraception is mandatory [27]. This renders it unsuitable for the large proportion of PCOS patients seeking fertility. Furthermore, it provides no benefit for the metabolic comorbidities of the syndrome. A comprehensive comparison of these pharmacological strategies, detailing their established mechanisms, clinical efficacy, and primary limitations, is provided in Table 1.

Current pharmacotherapy is largely fragmented. While agents like metformin and COCPs provide essential metabolic and symptomatic management, they often fail to comprehensively disrupt the self-perpetuating “Insulin Resistance—Hyperandrogenism” cycle or lack definitive long-term safety data for offspring. This underscores the urgent need for the precision-targeted strategies discussed in the following section.

**Table 1:** Overview of current pharmacological therapies for PCOS.

Drug Class	Mechanism of Action	Clinical Efficacy	Limitations and Challenges
COCPs	Dual Suppression: Progestin: Suppresses LH via HPO axis feedback. Estrogen: ↑ Hepatic SHBG → ↓ free testosterone [4].	First-line for menstrual regulation and cutaneous HA (acne, hirsutism). Reduces endometrial cancer risk.	Clinical Efficacy: Effective for menstrual regulation and cutaneous HA (acne, hirsutism). Metabolic: May exacerbate IR and endothelial dysfunction [11,12]. Safety: VTE risk, particularly in obesity/age > 35 [13,14,16,28].
Metformin	Insulin Sensitizer: Activates AMPK pathway [17]. ↓ Hepatic gluconeogenesis; ↑ peripheral glucose uptake [17].	Improves metabolic profiles and menstrual cyclicity. Synergistic: ↓ FAI and ↑ SHBG when added to COCPs [19].	Variable efficacy: Highly effective for metabolic traits, but inconsistent impact on cutaneous HA. Fertility: Inferior live birth rates vs. Letrozole; no benefit in IVF/ICSI [22]. Tolerability: Frequent GI distress reduces adherence. Offspring: Potential long-term cardiometabolic risk (MiG data) [23].
Letrozole	Aromatase Inhibitor: Blocks estrogen synthesis → releases HPO negative feedback → ↑ FSH secretion [24].	First-line for anovulatory infertility. Superior ovulation and live birth rates vs. Clomiphene [24].	Target limitation: Addresses anovulation without correcting metabolic/neuroendocrine drivers. Safety: Favorable safety profile with lower risk of OHSS compared to other agents [25]. Requires monitoring.
Spironolactone	Androgen Blockade: Competitive AR antagonist [26]. Inhibits 5α-reductase activity [26].	Effective for moderate-to-severe hirsutism refractory to COCP monotherapy.	Teratogenicity: Feminization of male fetus; mandates strict contraception [27]. Contraindication: Unsuitable for fertility-seeking patients. No metabolic benefit.

Abbreviations: AMPK: Adenosine Monophosphate-Activated Protein Kinase; AR: Androgen Receptor; FAI: Free Androgen Index; FSH: Follicle-Stimulating Hormone; GI: Gastrointestinal; HA: Hyperandrogenism; HPO: Hypothalamic-Pituitary-Ovarian; LH: Luteinizing Hormone; OHSS: Ovarian Hyperstimulation Syndrome; SHBG: Sex Hormone-Binding Globulin; VTE: Venous Thromboembolism. ↑ means “increase in”, ↓ means “decrease in”, → means “from x value to y value”.

## 3. Precision Targeted Therapies Based on Core Pathological Mechanisms

**Figure 1:**
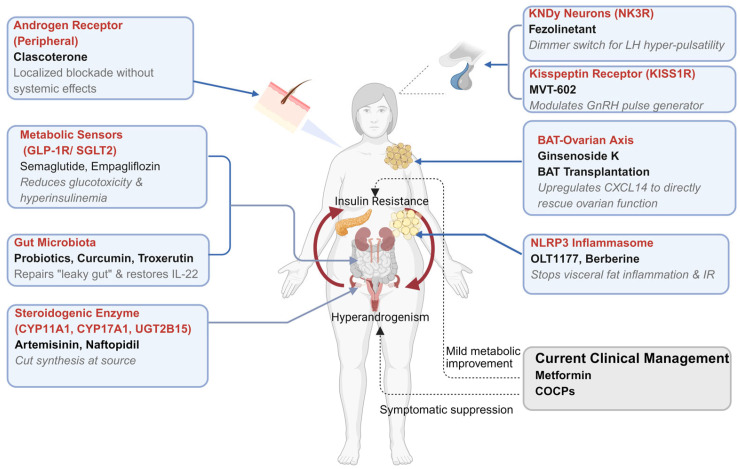
Schematic representation of the paradigm shift in PCOS pharmacotherapy: from symptomatic management to precision mechanism-based targeting. The central diagram illustrates the core pathophysiological “vicious cycle” driven by the interplay between insulin resistance and hyperandrogenism. (Grey Box and Dashed Lines) The bottom-right highlights the current standard of care (e.g., Metformin, COCPs), which exerts indirect, systemic modulation or symptomatic suppression. (Blue Boxes and Solid Lines) The callout boxes highlight emerging precision therapies that surgically intervene in specific pathological nodes discussed in this review. These novel strategies span multiple organ systems, including neuroendocrine modulation (KNDy neurons/NK3R, Kisspeptin receptor), metabolic sensors (GLP-1R/SGLT2, NLRP3 inflammasome), the reproductive axis (steroidogenic enzymes CYP11A1/CYP17A1), peripheral androgen blockade, and the novel BAT-ovarian axis and gut microbiota interventions. These targeted therapies aim to disrupt specific pathological nodes to achieve disease-modifying effects.

To address the limitations of traditional care, therapeutic development is shifting toward agents that precisely intervene in the specific pathological nodes of PCOS. At the heart of this pathology lies a self-reinforcing feedback loop: IR leads to compensatory hyperinsulinemia, which acts directly on ovarian theca cells to drive excess androgen production. Conversely, HA exacerbates visceral adiposity and metabolic dysfunction, further entrenching IR. Superimposed on this is a state of neuroendocrine dysregulation, characterized by high-frequency LH pulses that independently drive ovarian steroidogenesis. As illustrated in Figure 1, these emerging strategies aim to precisely disrupt the “IR–HA” vicious cycle at multiple organ levels—ranging from central neuroendocrine modulation and metabolic sensing to the direct inhibition of ovarian steroidogenesis.

### 3.1. Innovative Strategies Targeting Metabolic Dysregulation

#### 3.1.1. Glucagon-like Peptide-1 Receptor Agonists (GLP-1RAs)

Originally revolutionized the landscape of Type 2 Diabetes Mellitus (T2DM) and obesity management, GLP-1 receptor agonists (e.g., liraglutide, semaglutide) have rapidly emerged as a frontier in PCOS therapeutics. The rationale for this repurposing lies in the profound pathophysiological overlap between T2DM and PCOS: given that IR is a primary driver of ovarian androgen excess, agents that powerfully correct systemic metabolic dysfunction are hypothesized to indirectly restore ovarian homeostasis.

Unlike traditional insulin sensitizers like metformin, GLP-1RAs offer a pleiotropic mechanism of action that disrupts the core “IR-Hyperandrogenism” cycle at multiple levels. Centrally, they act on the hypothalamic proopiomelanocortin neurons and the mesolimbic reward system to suppress appetite and reduce cravings, leading to substantial weight loss. Peripherally, they enhance glucose-dependent insulin secretion and delay gastric emptying. Crucially for PCOS, emerging evidence suggests a direct effect: by mitigating hyperinsulinemia, these agents reduce the insulin-mediated stimulation of theca cell androgen production, thereby alleviating the hyperandrogenic milieu that suppresses ovulation [29,30].

Translating these mechanistic insights to the clinic, randomized trials have substantiated their superiority over standard care in reducing body weight and improving lipid profiles in obese PCOS phenotypes [31]. Innovation in this field is advancing towards “precision engineering”, exemplified by a novel GLP-1-estrogen conjugate that, in preclinical models, delivered targeted metabolic improvements while avoiding systemic reproductive toxicity [30]. However, clinical translation faces significant hurdles, primarily gastrointestinal adverse events that limit long-term adherence and the lack of clarity regarding whether reproductive benefits are independent of weight loss. Future research must prioritize RCTs to validate live birth rates and define the optimal therapeutic window—potentially positioning GLP-1RAs as a strategic “pre-treatment” to normalize metabolic health prior to ovulation induction.

#### 3.1.2. Sodium-Glucose Cotransporter 2 Inhibitors (SGLT2i)

Unlike insulin sensitizers that depend on intracellular signaling, SGLT2 inhibitors (e.g., empagliflozin, dapagliflozin) offer a distinct hemodynamic and metabolic approach by promoting glycosuria in the proximal renal tubules. The rationale for their use in PCOS extends beyond glycemic control; by reducing systemic hyperglycemia-induced hyperinsulinemia, these agents alleviate the chronic hyperinsulinemic drive on ovarian theca cells [32]. Furthermore, the caloric loss associated with glycosuria promotes reductions in visceral adiposity, indirectly dampening the inflammatory milieu that exacerbates hyperandrogenism [33,34]. Consistent with this rationale, emerging clinical data support this dual benefit. A randomized controlled trial comparing empagliflozin with metformin demonstrated that SGLT2 inhibition achieved superior improvements in anthropometric parameters and body composition [35]. However, clinical application is currently limited by the lack of long-term reproductive safety data and the theoretical risk of genital tract infections, which may be elevated in the PCOS population. Future studies must weigh these specific risks against metabolic benefits in non-diabetic PCOS cohorts.

#### 3.1.3. Targeting Brown Adipose Tissue (BAT)

Traditionally viewed solely as a thermogenic organ, Brown Adipose Tissue (BAT) has been redefined as a critical “metabolic endocrine organ” whose dysfunction is centrally implicated in PCOS [36]. The therapeutic logic here is transformative: it shifts focus from simply reducing white fat to activating brown fat. The mechanism involves a direct inter-organ communication pathway termed the “BAT-Ovarian Axis”. Activated BAT secretes specific “BATokines”, notably CXCL14, which acts directly on ovarian tissue to ameliorate dysfunction, suggesting that BAT’s influence extends beyond systemic metabolic improvements to direct reproductive regulation [37].

This paradigm is supported by robust translational evidence. Clinical observations confirm reduced BAT activity in women with PCOS [38,39]. Crucially, xenotransplantation of BAT in rodent models not only reversed insulin resistance but, more remarkably, restored estrous cyclicity and fertility [39,40]. Mechanistic studies further pinpointed that activation of BAT by compounds like Ginsenoside Compound K (CK) could rescue ovarian function via the CXCL14-mediated pathway [41].

Despite the compelling “BAT-Ovarian” theory, a significant translational gap remains due to the scarcity of active BAT in adult humans compared to rodents. The priority for future research is to identify safe pharmacological BAT activators viable for human use and to clinically validate the therapeutic relevance of CXCL14 levels in PCOS patients.

### 3.2. Precision Medications Targeting the Hypothalamic-Pituitary-Ovarian Axis

The central neuroendocrine hallmark of PCOS is characterized by a persistent increase in Gonadotropin-Releasing Hormone (GnRH) pulse frequency, which drives excessive Luteinizing Hormone (LH) secretion and subsequent ovarian androgen production. This pulsatility is governed by the KNDy neuronal network (Kisspeptin, Neurokinin B, and Dynorphin) located in the hypothalamic arcuate nucleus, effectively functioning as the “GnRH pulse generator”. Within this system, Kisspeptin acts as the primary driver triggering GnRH release, Neurokinin B (NKB) serves as the “accelerator”, and Dynorphin functions as the “brake”. In PCOS, this regulatory network is in a state of pathological hyperactivation. Consequently, targeted modulation of these specific upstream neuropeptides—rather than broad suppression of the HPO axis—may offer a precision strategy to restore neuroendocrine homeostasis.

#### 3.2.1. Kisspeptin Receptor (KISS1R) Agonists/Antagonists

As the proximal effector of GnRH secretion identified above, the Kisspeptin system presents a bimodal therapeutic opportunity. At the molecular level, Kisspeptin binds to the G-protein-coupled receptor 54 (KISS1R), activating the Gq/11 signaling cascade to trigger GnRH release [42,43]. Therapeutic strategies therefore diverge based on clinical goals: agonists can induce controlled LH surges for fertility treatment, while antagonists theoretically dampen the pathological LH hyperpulsatility that drives ovarian hyperandrogenism. In clinical settings, particularly in the realm of assisted reproductive technology, long-acting kisspeptin agonists, such as MVT-602, have been developed as physiological triggers for final oocyte maturation [44]. Evidence suggests they offer a safer alternative to hCG, significantly reducing the risk of OHSS while maintaining efficacy [45]. Conversely, for disease modification, preclinical studies indicate that specific inhibition of kisspeptin neurons can effectively reverse the hyperandrogenic phenotype in PCOS models by suppressing the neuroendocrine drive [46].

The primary translational challenge for antagonists lies in the “Goldilocks effect”: achieving partial suppression of LH pulses to control androgens without completely shutting down the reproductive axis (inducing amenorrhea) or causing hypoestrogenism. Developing orally active modulators with precise dosing regimens remains a critical priority for future clinical trials.

#### 3.2.2. Neuropeptide B/NK3 Receptor (NK3R) Antagonists

Targeting the “accelerator” component of the KNDy network offering a precision strike against neuroendocrine dysregulation. Unlike GnRH analogs that induce total gonadal suppression, NK3R antagonists (e.g., Fezolinetant) selectively block the excitatory signaling of Neurokinin B. This mechanism attenuates the pathologically high GnRH/LH pulse frequency to physiological levels, thereby lowering downstream testosterone production without abolishing basal estrogen secretion [47]. This represents a neuromodulatory approach rather than a total suppressive blockade.

Validating this mechanism in humans, Phase 2a clinical trials have validated this mechanism, showing that Fezolinetant effectively reduced LH and testosterone levels in women with PCOS [47]. Furthermore, emerging evidence suggests potential metabolic benefits mediated through adipocyte regulation, hinting at a dual therapeutic role beyond the HPO axis [48].

While promising, the primary hurdle is establishing long-term safety, particularly regarding potential liver enzyme elevations observed in other indications. The ultimate goal is to position NK3R antagonists as a non-contraceptive alternative for managing hyperandrogenism, filling a critical gap for women desiring fertility.

### 3.3. Strategies Targeting Androgen Synthesis and Signaling Pathways

#### 3.3.1. Targeting Steroidogenesis

Hyperandrogenism in PCOS is fueled by excessive ovarian biosynthesis. A breakthrough discovery published in *Science* (2024) [49] has identified Artemisinins—traditionally antimalarial drugs—as potent inhibitors of this process via a novel mechanism. Rather than acting as classic enzyme inhibitors, artemisinins target Lon Peptidase 1(LONP1) in the mitochondria to facilitate the degradation of CYP11A1. This “degradation-at-source” strategy provides a potent molecular brake on androgen production. This mechanism has been validated from bench to bedside. Artemisinin derivatives significantly reduced serum testosterone, improved polycystic morphology, and restored estrous cycle in rodent models. Promisingly, these findings have been mirrored in preliminary pilot studies in humans, showing concordant reductions in androgen levels. As a repurposed drug class, the immediate hurdle is establishing the optimal dosage for chronic PCOS management that balances efficacy with long-term safety. This discovery opens a new frontier for developing “degrader” therapies targeting specific steroidogenic enzymes.

#### 3.3.2. Regulation of the Androgen Receptor and Metabolism

Beyond synthesis, targeting the androgen receptor (AR) and metabolic clearance offers peripheral control of hyperandrogenism. A first-in-class topical AR antagonist have been developed that competitively inhibits DHT binding in the skin [50]. Its localized metabolism prevents systemic anti-androgenic side effects, addressing the specific clinical burden of hirsutism and acne [51,52]. The enzyme UGT2B15 is crucial for inactivating testosterone. In PCOS, its downregulation leads to androgen accumulation [53]. Novel agents like (R)- and (S)-nafepiride (NAF) work by upregulating UGT2B15 expression, effectively accelerating androgen clearance [54]. For Clascoterone, the limitation is its restriction to cutaneous symptoms without addressing systemic metabolic or reproductive dysfunction. For UGT2B15 modulators, the challenge remains in the early stage of development; proving clinical efficacy in human PCOS subjects is required to validate this metabolic clearance strategy.

### 3.4. Targeting the Immune-Inflammatory and Gut Axis

#### 3.4.1. NLRP3 Inflammasome Inhibitors

Chronic low-grade inflammation acts as a critical mediator amplifying the link between metabolic excess and insulin resistance (IR). The NLRP3 inflammasome serves as the molecular sensor in this process. In PCOS, metabolic danger signals (e.g., free fatty acids) trigger NLRP3 activation, leading to the release of IL-1β [55,56]. Mechanistically, IL-1β induces serine phosphorylation of insulin receptor substrate-1, directly impairing insulin signaling [57]. Therefore, inhibiting NLRP3 severs the link between metabolic stress and cellular insulin resistance.

Direct NLRP3 inhibitors like MCC950 and OLT1177 (Dapansutrile) have shown promise in reversing metabolic dysfunction in preclinical models [58]. While biologic agents targeting downstream IL-1β (e.g., Anakinra) are effective in diabetes [59,60], small-molecule NLRP3 inhibitors offer a more practical approach for chronic management.

Safety remains the paramount concern. MCC950 was halted due to hepatotoxicity, and while OLT1177 is safer, no dedicated PCOS trials exist [61,62]. Future development must focus on ensuring that immunomodulation does not compromise immune competence in this population.

#### 3.4.2. Targeting the Gut Microbiota-Bile Acid-IL-22 Axis

Gut dysbiosis is no longer seen as a bystander but as a driver of PCOS pathology through the “Gut-Bile Acid-IL-22” axis. Patients with PCOS exhibit an overabundance of *Bacteroides vulgatus*, which possesses high Bile Salt Hydrolase (BSH) activity [63].

This enzyme degrades conjugated bile acids (GDCA, TUDCA), which are essential for stimulating IL-22 secretion from intestinal immune cells (ILC3). The consequent deficiency in IL-22 leads to intestinal permeability, systemic inflammation, and insulin resistance [63,64]. Beyond bile acids, the ‘classical’ pathway involves the translocation of Lipopolysaccharides (LPS) from Gram-negative bacteria across a compromised intestinal barrier. This ‘metabolic endotoxemia’ triggers systemic inflammation via TLR4 activation, further exacerbating insulin resistance [65].

Therapeutic strategies aim to restore this axis: Probiotics/Synbiotics: Strains like *Lactobacillus* or *E. coli* Nissle 1917 have been shown to rebalance the microbiota, elevate IL-22 levels, and improve insulin sensitivity [66,67,68,69,70]. Emerging interest also focuses on postbiotics (bioactive compounds produced by food-grade microorganisms) as a potential strategy to modulate the gut environment with a favorable safety profile, particularly for immunocompromised individuals [71,72]. Fecal Microbiota Transplantation (FMT): Direct transplantation from healthy donors has reversed ovarian dysfunction and insulin resistance in rat models [73]. IL-22 Enhancers: Agents like Troxerutin have been identified to upregulate the secondary bile acids GDCA/TUDCA, thereby enhancing endogenous IL-22 production [74].

The complexity of the microbiome creates significant variability in treatment response. The primary bottleneck for FMT and probiotics is the lack of standardized protocols (strain specificity, dosage, duration). Future success depends on moving from generic “probiotic” supplementation to precision microbiome engineering based on individual patient profiles.

## 4. Natural Products: Multi-Target Intervention Strategies and Pharmacological Challenges

Given the multifaceted pathology of PCOS involving metabolism, inflammation, and endocrinology, single-target synthetic drugs often fail to achieve holistic control. Natural Products (NPs) offer a fundamentally different therapeutic paradigm: “Network Pharmacology”. Unlike the traditional ‘one drug, one target’ model, this approach conceptualizes disease as a perturbation of biological networks and seeks agents capable of restoring system-wide homeostasis through multi-component interventions. Unlike the “targeted therapeutic agents” discussed in Section 3, these agents act as “poly-pharmacological modulators”, simultaneously intervening in multiple nodes of the PCOS pathological network—concurrently improving insulin resistance, inhibiting androgen synthesis, and alleviating chronic inflammation—to restore systemic homeostasis. In this section, we focus on representative natural products—Berberine, Curcumin, Resveratrol, Quercetin, and Inositols—selected for their high ‘degree centrality’ in PCOS-target networks and their validated capacity to simultaneously modulate these metabolic, inflammatory, and endocrine pathways. However, a significant translational gap remains. The clinical efficacy of many natural phenols and alkaloids is currently compromised by poor oral bioavailability, rapid hepatic metabolism, and low plasma stability. While advanced pharmaceutical engineering (e.g., nanoparticles, phytosomes) offers a theoretical solution to enhance systemic exposure, the high manufacturing costs and technical complexity of these formulations currently limit their widespread accessibility. Consequently, most of these agents remain positioned as dietary supplements rather than standardized pharmaceutical prescriptions.

### 4.1. Berberine (BBR): A Natural AMPK Activator

Berberine stands as the quintessential multi-target agent. It acts as a potent AMPK activator (similar to metformin), promoting the PI3K/AKT pathway and GLUT4 translocation to reverse systemic insulin resistance [75,76].

Crucially, unlike metformin, BBR exhibits a direct anti-androgenic effect by inhibiting the expression of key steroidogenic enzymes (CYP17A1, StAR) and suppressing Aldo-Keto Reductase (AKR1C3) activity in ovarian theca cells [77,78,79]. This multi-pronged mechanism translates into broad clinical benefits. In PCOS-IR models, BBR has been shown to simultaneously improve insulin sensitivity, lipid profiles, and hyperandrogenism. Corroborating these animal data, clinical studies suggest efficacy comparable to metformin with potentially superior lipid-lowering effects [80]. The limitation of BBR is its poor oral bioavailability due to intestinal efflux transporters (P-glycoprotein) and frequent gastrointestinal side effects. Future formulations, such as nano-micelles or co-administration with absorption enhancers, are essential to unlock its full clinical potential.

### 4.2. Curcumin: Gut Barrier Repair and Anti-Inflammatory Effects

“Metabolic Endotoxemia” stemming from a “leaky gut” is a key driver of chronic inflammation in PCOS [81,82]. This concept aligns with the ‘Dysbiosis of Gut Microbiota (DOGMA)’ theory first proposed by Tremellen and Pearce [83], which posits that intestinal permeability drives the inflammatory phenotype. Curcumin offers a unique dual-intervention strategy targeting the axis as follows. Upstream, it activates PPARγ to upregulate tight junction proteins (zonula occludens-1, occludin), effectively “sealing” the intestinal epithelial barrier against LPS leakage Downstream, it directly inhibits the TLR4/NF-κB signaling cascade, blocking the cytokine storm induced by circulating endotoxins [84].

Despite its low solubility, advanced delivery systems have validated this mechanism. Preclinical studies using Nanocurcumin demonstrated significant reductions in oxidative stress markers, TNF-α, and insulin levels [85].

Furthermore, Self-nanoemulsifying Drug Delivery Systems (SEDDSs) co-delivering curcumin have shown superior glycemic control compared to crude mixtures [86]. The primary bottleneck is its extremely low oral bioavailability and rapid metabolism. Clinical translation is strictly dependent on the success of novel drug delivery technologies (e.g., liposomes, nanoparticles) to ensure therapeutic plasma concentrations.

### 4.3. Resveratrol: SIRT1 Activator and Direct Anti-Androgenic Effects

Resveratrol mimics the benefits of calorie restriction by activating SIRT1, a master regulator of cellular energy metabolism. Systemically, activation of the SIRT1/AMPK axis improves glucose homeostasis [87]. Uniquely at the ovarian level, resveratrol exerts a direct inhibitory effect on ovarian theca cells by downregulating CYP17A1 activity, thereby blocking the conversion of progesterone to androgens at the source [88,89]. Additionally, it promotes oocyte quality by repairing transzonal projections (TZPs), facilitating bidirectional communication between oocytes and granulosa cells [90]. Supporting these mechanistic benefits, clinical utility is supported by a double-blind randomized controlled trial showing that 1500 mg of resveratrol daily significantly reduced serum total testosterone and DHEAS levels in women with PCOS [88]. Further reviews confirm its ability to improve ovarian morphology and alleviate endoplasmic reticulum stress [91]. Rapid metabolism leads to a short half-life, necessitating high daily doses for clinical effect. Future research should focus on more stable analogs or synergistic combinations (e.g., with inositols) to enhance efficacy at lower doses.

### 4.4. Quercetin: A Multifaceted Modulator of Inflammation and Metabolism

Quercetin targets the oxidative stress and inflammatory component of PCOS through a broad-spectrum approach. As a potent anti-inflammatory agent, it inhibits the NLRP3 inflammasome and NF-κB pathway in macrophages, cutting off the production of pro-inflammatory cytokines like IL-1β [92,93,94,95,96,97]. In terms of antioxidant defense, it upregulates the Nrf2/Thioredoxin system to combat granulosa cell apoptosis [98,99]. Concurrently acting on metabolic and endocrine fronts, it activates AMPK to improve glucose uptake and inhibits ovarian steroidogenic enzymes (17β-HSD, CYP17A1) to lower androgen output [100,101,102]. In vitro and in vivo studies confirm its ability to protect ovarian function against toxic insults (e.g., cadmium) and improve metabolic parameters. Similarly to other flavonoids, quercetin suffers from poor water solubility and rapid hepatic/intestinal inactivation (glucuronidation) (<5% bioavailability). Developing reliable nanoparticle delivery systems is the prerequisite for its transition from a dietary supplement to a therapeutic agent.

### 4.5. Inositols: Natural Insulin Sensitizers and Ovulation Restorers

Inositols, particularly myo-inositol (MI) and D-chiro-inositol (DCI), serve as critical secondary messengers in insulin signal transduction, addressing both metabolic and reproductive dysfunctions. Functionally, MI acts to facilitate cellular glucose uptake and supports FSH signaling, directly promoting follicle maturation. Concurrently, DCI mediates insulin-induced androgen synthesis and glycogen storage. Clinical evidence strongly supports their therapeutic utility; randomized controlled trials and meta-analyses have demonstrated that inositol supplementation significantly improves homeostatic model assessment of insulin resistance (HOMA-IR) and restores spontaneous ovulation, leading to their endorsement in the 2023 International Evidence-based Guidelines [4,103]. However, a unique translational challenge exists: the “Ovarian Paradox”. While systemic tissues in PCOS may be resistant to insulin, the ovary remains sensitive, often exhibiting pathologically high epimerase activity that converts MI to DCI too rapidly. Consequently, efficacy is strictly dependent on the formulation; high doses of DCI alone may paradoxically worsen hyperandrogenism. Therefore, future therapeutic success depends on adhering to the physiological 40:1 (MI:DCI) ratio to mimic plasma homeostasis, rather than generic inositol supplementation. The specific multi-target mechanisms, therapeutic effects, and current translational bottlenecks of these representative natural products are summarized in Table 2.

## 5. Conclusions

The therapeutic landscape for PCOS is rapidly evolving from a “one-size-fits-all” approach of symptom management to a new era of pathophysiology-driven precision medicine. As synthesized in this review, this transformation is characterized by the emergence of agents that do not merely suppress symptoms but actively interrogate and correct the underlying dysregulation of the “IR-HA” axis. Transitioning from symptomatic relief to disease modification, the limitations of current standard care—COCPs and metformin—underscore the urgent need for innovation. Emerging precision therapies are beginning to bridge this gap. Metabolic agents like GLP-1 receptor agonists and SGLT2 inhibitors are redefining treatment goals by targeting systemic glucotoxicity and obesity, potentially serving as essential “pre-treatment” strategies to optimize reproductive outcomes. Simultaneously, discoveries such as the “BAT-Ovarian Axis” and the LONP1-mediated degradation of CYP11A1 by Artemisinins have unveiled entirely new druggable targets, proving that intervention can be achieved not just at the receptor level, but at the organ-communication and protein-stability levels. Furthermore, the development of NK3R antagonists offers a rheostatic approach to neuroendocrine control, promising effective androgen suppression without the total gonadal shutdown associated with traditional hormonal therapies. Complementing these high-precision tools, the strategic value of “Network Pharmacology” offers a distinct advantage. Agents like Berberine, Resveratrol, and Curcumin demonstrate that moderate, simultaneous modulation of multiple pathways—metabolism, inflammation, and gut barrier function—can achieve holistic restoration of systemic homeostasis, often with a favorable safety profile suitable for long-term maintenance.

## 6. Future Perspectives

Despite this promise, navigating the translational gap remains a critical challenge. The transition from preclinical excitement to clinical reality requires addressing the “Intergenerational Safety” hurdle. As highlighted by the long-term data on metformin, the safety of novel agents (e.g., GLP-1RAs, NLRP3 inhibitors) during the periconceptional period and their impact on offspring metabolic health must be rigorously established before widespread adoption. Additionally, the heterogeneity of PCOS mandates a shift towards biomarker-driven clinical trials.

Beyond safety, the ultimate realization of precision medicine hinges on stratifying patients by specific phenotypes. Metabolic-Predominant Phenotypes (characterized by marked insulin resistance and obesity) are prime candidates for metabolic regulators (e.g., GLP-1RAs, SGLT2i), whereas Reproductive-Predominant Phenotypes (driven by neuroendocrine dysfunction and high LH pulse frequency) may benefit most from neuroendocrine modulators (e.g., NK3R antagonists). This approach facilitates a shift from trial-and-error to targeted intervention.

In summary, the future of PCOS pharmacotherapy lies in the integration of precision targets and the restoration of systemic homeostasis. By moving beyond the suppression of symptoms to the correction of core mechanisms, the next generation of therapeutics holds the potential to break the self-perpetuating cycle of PCOS, offering women not just temporary relief, but long-term health and fertility preservation.

## Figures and Tables

**Table 2:** Mechanisms and Translational Challenges of Representative Natural Products (NPs) for PCOS.

Natural Products	Key Mechanisms	Therapeutic Effects	Translational Challenges
Berberine	AMPK Activator: Promotes PI3K/AKT pathway and GLUT4 translocation [75,76]. Enzyme Inhibition: Suppresses ovarian CYP17A1, StAR and AKR1C3 activity [77,78,79].	Metabolic: Reverses systemic IR; improves lipid profiles [80]. Endocrine: Directly reduces androgen synthesis and serum testosterone.	Low Bioavailability: Substrate of P-glycoprotein efflux transporters. Side Effects: Frequent GI distress limits adherence.
Curcumin	Gut-Systemic Axis: Activates PPAR-γ to upregulate tight junction proteins [81,82]. Anti-inflammatory: Blocks TLR4/NF-κB cascade [84].	Gut Health: Repairs “leaky gut” and prevents metabolic endotoxemia. Systemic: ↓ Cytokine storm and oxidative stress markers [85].	Poor Pharmacokinetics: Extremely low solubility and rapid systemic elimination. Requires advanced delivery systems (e.g., SEDDS, nanoparticles) [86].
Resveratrol	SIRT1 Activation: Mimics calorie restriction [87]. Ovarian Direct Action: Downregulates CYP17A1 [88,89] and repairs TZPs [90].	Metabolic: Improves glucose homeostasis Reproductive: ↓ DHEAS/Testosterone levels; ↑ oocyte quality and morphology [91].	Rapid Metabolism: Short half-life necessitates high daily doses (e.g., 1500 mg). Variable clinical efficacy dependent on dosage formulation.
Quercetin	Inflammasome Inhibition: Targets NLRP3 and NF-κB [92,93,94,95,96,97]. Antioxidant: Upregulates Nrf2 pathway [98,99]. Steroidogenesis: Inhibits 17β-HSD and CYP17A1 [100,101,102].	Cellular Protection: Combats granulosa cell apoptosis and oxidative injury. Endocrine: Lowers androgen output.	Low Bioavailability (<5%): Rapid hepatic/intestinal glucuronidation. Transition from dietary supplement to therapeutic agent requires stable formulations.
Inositols (MI/DCI)	Insulin second messengers; MI improves FSH signaling; DCI mediates insulin-induced androgen synthesis.	Metabolic: Improves insulin sensitivity and glucose tolerance. Reproductive: Restores spontaneous ovulation and improves oocyte quality.	Ratio Specificity: Efficacy is highly dependent on the optimal MI:DCI ratio (40:1); DCI overdose may paradoxically worsen androgen levels.

Abbreviations: AKR1C3: Aldo-Keto Reductase Family 1 Member C3; AMPK: Adenosine Monophosphate-Activated Protein Kinase; CYP17A1: Cytochrome P450 17A1; GLUT4: Glucose Transporter Type 4; IR: Insulin Resistance; NF-κB: Nuclear Factor Kappa-Light-Chain-Enhancer of Activated B Cells; NLRP3: NLR Family Pyrin Domain Containing 3; Nrf2: Nuclear Factor Erythroid 2-Related Factor 2; PI3K, Phosphoinositide 3-Kinase; PPAR-γ: Peroxisome Proliferator-Activated Receptor Gamma; SEDDS: Self-Nanoemulsifying Drug Delivery Systems; SIRT1: Sirtuin 1; StAR: Steroidogenic Acute Regulatory Protein; TLR4: Toll-Like Receptor 4; TZPs: Transzonal Projections; ↑ means increase, ↓ means decrease.

## Data Availability

No new data were created or analyzed in this study. Data sharing is not applicable to this article.

## Abbreviations

17β-HSD—17β-Hydroxysteroid Dehydrogenase; AKR1C3—Aldo-Keto Reductase Family 1 Member C3; AMPK—Adenosine Monophosphate-Activated Protein Kinase; AR—Androgen Receptor; BAT—Brown Adipose Tissue; BBR—Berberine; CK—Ginsenoside Compound K; HPO Axis—Hypothalamic-Pituitary-Ovarian Axis; COCPs—Combined Oral Contraceptives; CXCL14—C-X-C Motif Chemokine Ligand 14; CYP11A1—Cholesterol Side-Chain Cleavage Enzyme; CYP17A1—17α-Hydroxylase/17,20-Lyase; FMT—Fecal Microbiota Transplantation; GDCA—Glycodeoxycholic Acid; GLP-1RAs—Glucagon-Like Peptide-1 Receptor Agonists; GLUT4—Glucose Transporter Type 4; GnRH—Gonadotropin-Releasing Hormone; HA—Hyperandrogenism; IL-1β—Interleukin-1 Beta; IL-22—Interleukin-22; IR—Insulin Resistance; KISS1R—Kisspeptin Receptor; LH—Luteinizing Hormone; LONP1—Lon Peptidase 1; LPS—Lipopolysaccharide; MiG—Metformin Interventions in Gestation; NF-κB—Nuclear Factor Kappa-Light-Chain-Enhancer of Activated B Cells; NK3R—Neurokinin 3 Receptor; NKB—Neurokinin B; NLRP3—NLR Family Pyrin Domain Containing 3; NPs—Natural Products; Nrf2—Nuclear Factor Erythroid 2-Related Factor 2; OHSS—Ovarian Hyperstimulation Syndrome; PCOS—Polycystic Ovary Syndrome; PI3K—Phosphoinositide 3-Kinase; PPAR-γ—Peroxisome Proliferator-Activated Receptor Gamma; RCTs—Randomized Controlled Trials; SGLT2i—Sodium-Glucose Cotransporter 2 Inhibitors; SHBG—Sex Hormone-Binding Globulin; SIRT1—Sirtuin 1; StAR—Steroidogenic Acute Regulatory Protein; T2DM—Type 2 Diabetes Mellitus; TLR4—Toll-Like Receptor 4; TNF-α—Tumor Necrosis Factor-Alpha; TUDCA—Tauroursodeoxycholic Acid; TZPs—Transzonal Projections; UGT2B15—UDP Glucuronosyltransferase Family 2 Member B15; VTE—Venous Thromboembolism.
